# Ergot alkaloid intoxication in perennial ryegrass (*Lolium perenne*): an emerging animal health concern in Ireland?

**DOI:** 10.1186/2046-0481-67-21

**Published:** 2014-09-25

**Authors:** Mary J Canty, Ursula Fogarty, Michael K Sheridan, Steve M Ensley, Dwayne E Schrunk, Simon J More

**Affiliations:** 1 Centre for Veterinary Epidemiology and Risk Analysis (CVERA), Veterinary Sciences Centre, University College Dublin, Belfield, Dublin 4, Ireland; 2 Department of Agriculture, Food and the Marine, Agriculture House, Kildare St, Dublin 2, Ireland; 3 Irish Equine Centre, Johnstown, Naas, Co. Kildare, Ireland; 4 College of Veterinary Medicine, Iowa State University, Ames, IA 2011, USA

**Keywords:** Ergot alkaloids, Intoxication, Perennial ryegrass, Animal health, Ireland

## Abstract

Four primary mycotoxicosis have been reported in livestock caused by fungal infections of grasses or cereals by members of the *Clavicipitaceae* family. Ergotism (generally associated with grasses, rye, triticale and other grains) and fescue toxicosis (associated with tall fescue grass, *Festuca arundinacea*) are both caused by ergot alkaloids, and referred to as ‘ergot alkaloid intoxication’. Ryegrass staggers (associated with perennial ryegrass *Lolium perenne*) is due to intoxication with an indole-diperpene, Lolitrem B, and metabolites. Fescue-associated oedema, recently described in Australia, may be associated with a pyrrolizidine alkaloid, N-acetyl norloline.

Ergotism, caused by the fungus *Claviceps purpurea*, is visible and infects the outside of the plant seed. Fescue toxicosis and ryegrass staggers are caused by *Neotyphodium coenophalium* and *N. lolii*, respectively. Fescue-associated oedema has been associated with tall fescue varieties infected with a specific strain of *N. coenophialum* (AR542, Max P or Max Q). The name *Neotyphodium* refers to asexual derivatives of *Epichloë* spp., which have collectively been termed the epichloë fungi. These fungi exist symbiotically within the grass and are invisible to the naked eye.

The primary toxicological effect of ergot alkaloid involves vasoconstriction and/or hypoprolactinaemia. Ingestion of ergot alkaloid by livestock can cause a range of effects, including poor weight gain, reduced fertility, hyperthermia, convulsions, gangrene of the extremities, and death. To date there are no published reports, either internationally or nationally, reporting ergot alkaloid intoxication specifically associated with perennial ryegrass endophytes. However, unpublished reports from the Irish Equine Centre have identified a potential emerging problem of ergot alkaloid intoxication with respect to equines and bovines, on primarily perennial ryegrass-based diets. Ergovaline has been isolated in varying concentrations in the herbage of a small number of equine and bovine farms where poor animal health and performance had been reported. Additionally, in some circumstances changes to the diet, where animals were fed primarily herbage, were sufficient to reverse adverse effects. Pending additional information, these results suggest that Irish farm advisors and veterinarians should be aware of the potential adverse role on animal health and performance of ergot alkaloids from perennial ryegrass infected with endophytic fungi.

## Introduction

### Mycotoxicosis associated with grasses and cereals

Four primary mycotoxicosis in livestock associated with fungal infections of grasses or cereals by members of the *Clavicipitaceae* family have been reported in the international literature (Table 1), including:

• *Ergotism,* principally associated with ergotamine, which is an ergot alkaloid toxin produced by the external fungus *Claviceps purpurea* typically found on the seed heads in grass, rye and other cereals;

• *Fescue toxicosis (including fescue foot),* principally associated with ergovaline, the ergot alkaloid toxin produced by the internal (or endophyte) fungus *Neotyphodium* (previously *Acremonium*) *coenophialum* found in tall fescue (*Festuca arundinacea*) grass;

• *Ryegrass staggers,* principally associated with Lolitrem B, an indole-diterpene toxin (not an ergot alkaloid toxin), and metabolites produced by the endophyte fungus *N. lolii* found in perennial ryegrass (*Lolium perenne*); and

• *Fescue-associated oedema*, recently described in Australia in horses grazing pastures of tall fescue *(F. arundinacea)* carrying a specific strain of *N. coenophialum* (AR542; Max P or Max Q) that does not produce ergovaline. A pyrrolizine alkaloid, N-acetyl norloline, may be responsible [1].

**Table 1:** **Mycotoxicoses in cattle and horses associated with fungal infections of grasses or cereals by members of the *Clavicipitaceae *** **family, based on data from Radostits **[2]**and Mostrom and Jacobson **[3]

**Fungus**	**Mycotoxin**	**Disease**	**Clinical signs and pathogenesis**
**[Grass & cereal]**			
*Neotyphodium lolii*	Lolitrems (Lolitrem B), an indole-diterpene toxin	Ryegrass staggers	When disturbed gross incoordination, falling hypersensitivity. Functional derangement of nervous tissue function. No histological lesions
[Perennial ryegrass (*Lolium perennae*)]			
*Neotyphodium coenophialum*	Ergovaline, an ergot alkaloid	Fescue toxicosis	Low milk yield or weight gain, hypersalivation, seek shade. Depression of blood prolactin concentrations
[Tall fescue (*Festuca arundinaceae*)]		Fescue foot	Loss of tail switch, distal limbs, tail tip gangrene. Local vasoconstriction restricts blood supply
[Perennial ryegrass *(Lolium perennae)*]		Prolonged gestation	Long gestation, dystocia, abortion, stillbirth, agalactia. Vasoconstriction cause placental edema, reducing circulating prolactin
		
*Claviceps purpurea*	A range of ergot alkaloids, principally ergotamine, but also ergocristine, ergosine, ergocorine and ergocryptine	Ergotism	Lameness, gangrene of lower limbs, ear tips, loss of tail switch. Arteriolar spasm causes deficient blood supply body parts
[Cereals, rye, triticale, grains, grasses]		Hyperthermia	Hyperthermia, salivation, dyspnea. Reduced blood supply to skin reduces heat loss

Mycotoxicosis from grasses and cereals is a recognized animal health issue (Table 1). For example, there are numerous reports of fescue toxicosis from the USA [4,5], New Zealand [6-8] and Australia [1,6]. After taking account of impact on equines and on both small and large ruminants, it is estimated that the combined losses due to ergot alkaloid intoxication of animal feed in the USA are likely to exceed $1 billion annually [9]. In Europe, intoxications of livestock with ergot toxins has largely been associated with cereals [10]. There have been reports of ergot poisoning in both Ireland and England due to *Claviceps purpura*[11,12]. Lameness and mycotoxicoses are difficult to differentiate from diseases with similar epidemiological, clinical, clinicopathological and histopathological profiles, noting that the fungal agent may be invisible to the eye, for example *N. coenophalium*[2], and generally relies on evaluation of a representative sample of the suspect feed(s) consumed by the livestock.

**Figure 1:**
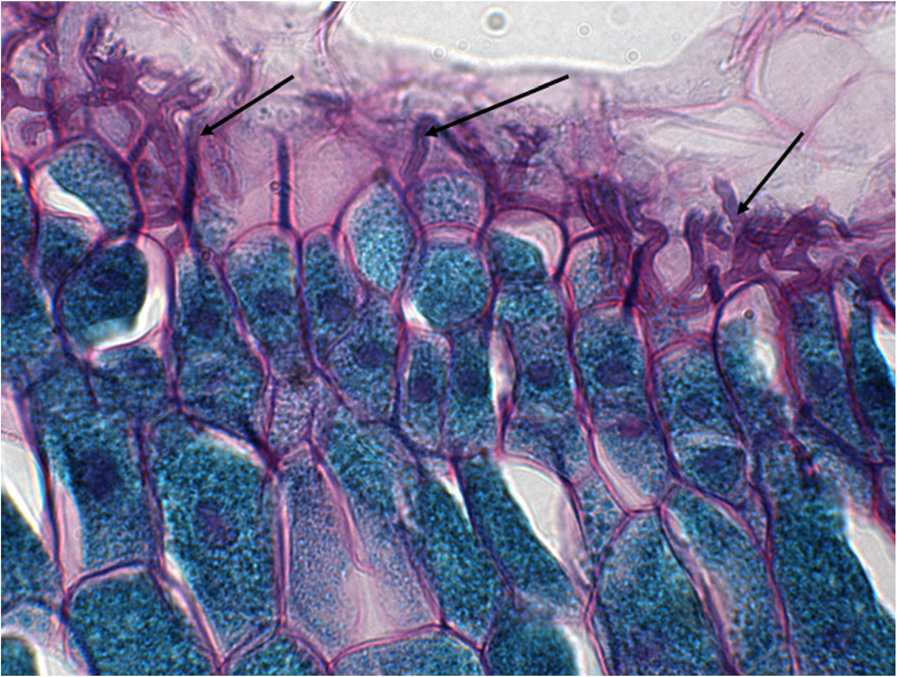
**Perennial ryegrass infected with endophytes, as indicated by the black arrows.** Periodic acid-Schiff stained 3 μm section, 1000× oil immersion.

**Figure 2:**
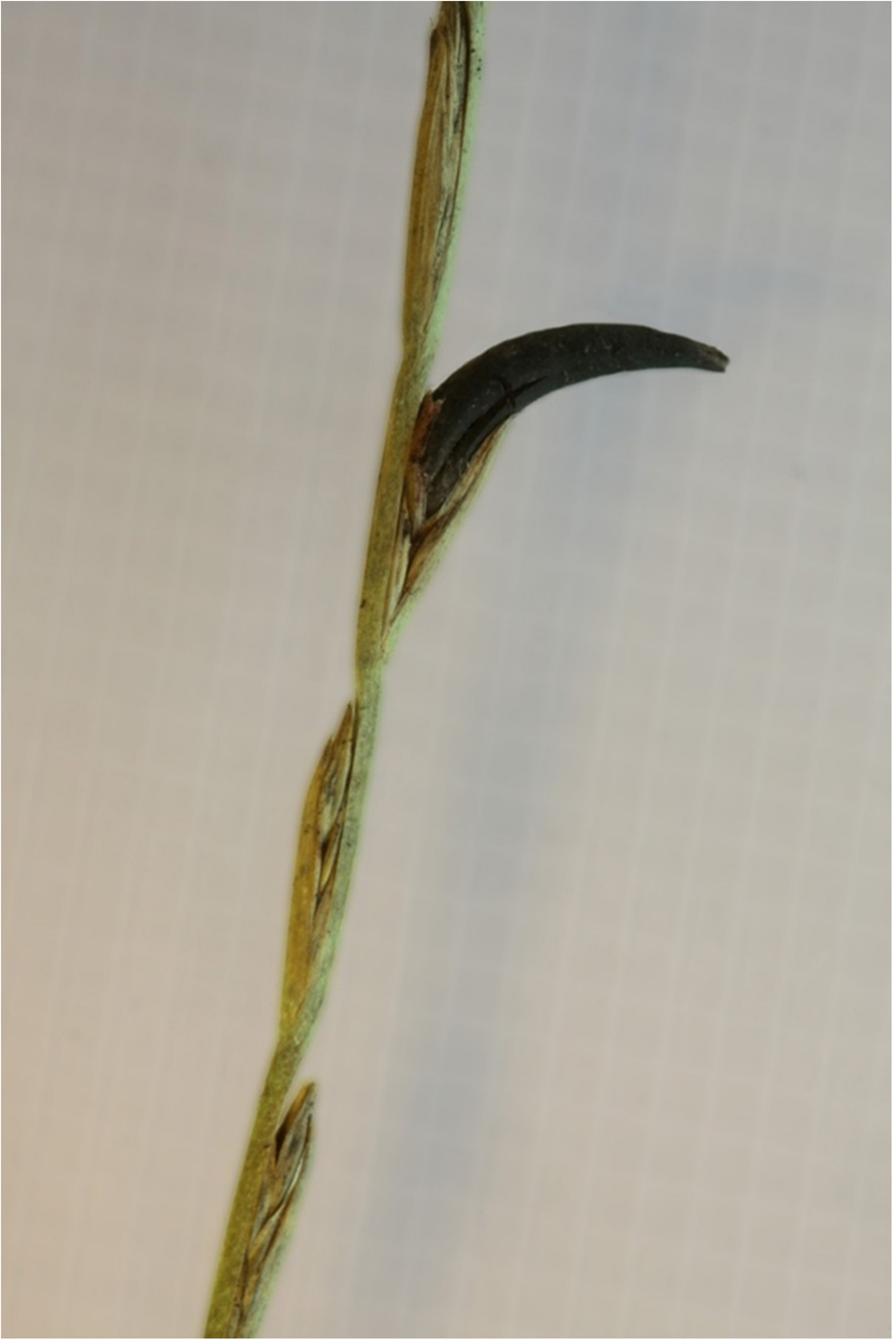
**Externally visible parasitic fungus *Claviceps purpurea *** **on the plant/seed.**

The name *Neotyphodium* refers to asexual derivatives of *Epichloë* spp., which have collectively been termed the epichloë fungi [13] or *Epichloë/Neotyphodium* spp. [14]. A change in nomenclature, to align these two families, has recently been proposed [15]. They are symbiotic fungi (endophytes) that grow within cool season grasses, in an apparent co-evolutionary relationship [14]. *Neotyphodium* spp. are invisible to the naked eye (Figure 1). In contrast, *C. purpurea* is externally visible on the plant and seeds (Figure 2) and therefore easier to associate with clinical signs in affected grazing livestock. These fungi are all members of the same fungal family, *Clavicipitaceae*. Within this fungal family, the fungi are divided into those that reproduce sexually, including *C. purpurea* and *Epichloë* spp., and those that are asexual (e.g. *N. coenophialum* and *N. lolii*). *Epichloë* spp. can reproduce sexually by forming stromata, whereas *Neotyphodium* endophytes are always transmitted by hyphae growing into the developing seeds of maternal host plants [16].

Grasses naturally cohabitate with numerous microorganisms including fungal endophytes, which are important to the ecological fitness and species diversity of the grass. The fungal endophytes benefit from inhabiting the grass’s interior because this is a protected area, with little competition from other microorganisms, and a reliable source of nutrition [17]. In turn, endophyte infection confers several ecological benefits to infected plants, including resistance to invertebrate and vertebrate herbivory. It may also facilitate enhanced growth, mineral uptake and resistance to drought [17-20], thereby acting as a biocontrol agent resulting in improved plant persistence [21].

This article focuses on ergot alkaloid intoxication in farmed animals, with two objectives. Firstly, we present current knowledge based on a review of the international scientific literature. Secondly, we summarise data that are currently available in Ireland, highlighting knowledge gaps and presenting recommendations.

## Review

### Ergot alkaloids

Ergot alkaloids are complex compounds produced by certain fungi. They are secondary metabolites most of which have been isolated from fungal species belonging to the family of *Clavicipitaceae*. They include *C. purpurea*, which occurs predominantly on rye, wheat and barley, and *Neotyphodium* species, which infect forages and turf grasses, including perennial rye grass and tall fescue (*F. arundinacea*) [10,22], and cereals. The common structural feature of natural ergot alkaloid is the ergoline ring methylated on nitrogen N-6 and variously substituted on C-8 [23,24]. Depending on the substitution on C-8, ergot alkaloids are classified into four groups: clavines, lysergic acid, simple lysergic acid amides, and ergopeptines [23]. In this review, with a focus on the causes of ergotism and fescue toxicosis, it is primarily the ergopeptines that are of interest. This is a complex area, noting the considerable variation that is present both between and even within *Neotyphodium* species with respect to the alkaloids produced [14]. Further, different ergot alkaloids have different biological activities. *N. coenophialum* alone produces over 30 ergot alkaloids, including 17 that affect livestock. Nonetheless, ergovaline is the primary ergopeptide produced by the *Neotyphodium* spp. [19,25] and the principal toxin associated with the different forms of fescue toxicosis. Ergovaline is thought to be the most physiologically active ergot alkaloid produced by *N. coenophialum*[22]. It has been demonstrated that ergovaline is approximately 1,000 times more potent and 5 times more efficacious in causing vasoconstriction than lysergic acid [26,27]. Similarly, *C. purpurea* produces many ergopeptides, the most prominent toxins amongst them being ergotamine, ergocristine, ergosine, ergocryptine and ergocornine [22,24].

Although there are differences in the primary ergopeptide produced by *Neotyphodium* spp. and *C. purpurea*, the mode of action of these ergopeptides is similar and the clinical presentation of toxicity in animals can be indistinguishable. Any clinical differences between fescue toxicosis and ergotism, if observed, is likely to be due to the higher ergopeptine alkaloid concentrations in ergot (*C. purpurea*) compared with fescue endophytes, and the tendency for a longer duration of exposure to fescue endophytes [28]. In the scientific literature, fescue toxicosis and ergotism are frequently considered as distinct intoxications, reflecting differences in fungal sources. However, both are the consequences of the adverse effects of ergot alkaloids, and have been collectively referred to as ‘ergot alkaloid intoxication’ [3].

## Mode of action of ergot alkaloids

The biological activity of the ergot alkaloids in animal systems is largely due to the similarity of the ergoline ring structure to biogenic amines such as serotonin, dopamine, noradrenaline, and adrenaline. The structural similarity allows many of the ergot alkaloids to bind biogenic amine receptors and to elicit such effects as decreased serum prolactin and vasoconstriction [9]. The primary pharmaceutical actions of ergot alkaloid, whether of endophytic or ergot origin, involves vasoconstriction and/or hypoprolactinaemia. Vasoconstriction is association with D_1_-dopaminergic receptor inhibition and partial agonism of the α_1_-adrenergic and serotonin receptors. Hypoprolactinaemia results from the stimulation of lactotrophic D_2_-dopamine receptors in the anterior pituitary and inhibition of probating secretion. In ruminants, vasoconstrictive effects predominate and cause gangrenous and hyperthermic forms of ergot alkaloid intoxication, with the gangrenous syndrome generally occurring in low environmental temperatures. It results in diminished blood flow to the extremities, lameness and eventually dry gangrene. Prolactin plays a diverse role in lactogenesis, steroidogenesis and various reproductive pathways, and the reproductive form of ergot alkaloid intoxication is characterized by decreased milk production, abnormal progestagen metabolism, delayed parturition, and other reproductive abnormalities, including subfertility. Horses are more susceptible to reproductive effects than ruminants, as ruminants produce a placental lactogen during pregnancy [28].

Ergot alkaloid intoxication results in disruption of several physiological systems, relating to reproduction, growth, cardiovascular function, and the signs of these disruptions vary with severity. Ruminant livestock are generally less affected than non-ruminants and hindgut fibre digesting livestock [9]. Susceptibility of livestock to ergotism seems to vary with species, breed, age, gender, and physiologic state [3], with other stressors and management practices also playing a contributory role in the pathogenesis of impaired reproductive function. There is some evidence that ergopeptines, including ergovaline, bio-accumulate in body fat/lipid stores. However, very little is known about the distribution of ergot alkaloids in the tissue of grazing livestock. This lack of knowledge may in part be due to technical constraints in the sensitivity and selectivity of the analytical methods available for the detection of these alkaloids generally [9].

## Animal performance and ergot alkaloids

The effects of ingestion of ergot alkaloid will vary substantially in accordance with the fungal source and the level of infestation within the grass or cereal. Ingestion of ergot alkaloid by livestock can cause a range of effects from reduced performance to acute clinical signs. These include poor weight gain, reduced fertility, hyperthermia, convulsions, gangrene of the extremities, and death [3,28]. Further, reduced feed intake, dry matter digestibility, nitrogen retention, average daily gain, serum prolactin and elevated rectal temperatures [29,30] and reduced growth and prolactin in heifers [4,5] have been reported in steers and heifers, respectively, that were fed endophyte-infected tall fescue. In relatively cool temperatures, Holstein cows fed a total mixed ration with fescue hay containing either low (45 μg/kg DM) or high (782 μg/kg DM) concentrations of ergovaline showed similar intake and milk yields, but milk fat and milk protein were reduced in the high ergovaline diet [31]. Conversely, a 4.6 L reduction in milk production of Holstein-Friesian cows was associated with high concentrations of ergovaline in ryegrass silage (total silage ergovaline concentrations of 1.78 μg/g) [7]. Cows also had elevated milk somatic cell counts and reduced reproductive performance. It has been proposed that cows under metabolic stress during the transition period (from drying off through to early lactation) may be more sensitive to the effects of feed contaminated with mycotoxin, amongst other things [32]. No adverse effect on pregnancy rate and embryonic loss were reported in beef cows that were grazing endophyte-infected tall fescue [33]. However, it was reported that less oestrous activity was observed following synchronization treatment in weanling heifers grazing high endophyte pastures [4]. Progesterone concentrations in these heifers suggested a higher incidence of luteal dysfunction. Despite this, pregnancy rates were similar to heifers fed low endophyte-infected tall fescue.

Reproductive disorders arising from exposure to ergot alkaloids are more common and more dramatic in horses than ruminants, and particularly among late-gestational and early post-parturient mares [22]. These disorders include failure to come into heat, early-term abortions, prolonged pregnancies, dystocia, retained placentas, poor udder development with little or no milk production, and poor foal survival. Mares removed from endophyte-infected pastures one month prior to foaling usually recover and have normal foals, however, milk production may be decreased [19]. The effect on stallions is less pronounced. A preliminary study of breeding stallions given feed containing infected fescue seed showed that sperm motility, number morphology and sperm morphology were not different when compared with control animals [34]. Similarly, a study in ram lambs fed endophyte-infected fescue seed (34% of the diet, 4.8 μg/g ergovaline) showed that scrotal temperature, scrotal circumference, semen volume, percent sperm motility, and percent normal sperm were not different compared with control rams [35].

### Threshold concentrations for ergot alkaloid affecting animal health

Diagnostic testing for ergot alkaloid intoxication (fescue toxicosis and ergotism) includes the use of high performance liquid chromatography (HPLC) to determine the concentrations of ergovaline, ergotamine, ergocristine, ergosine, ergocornine and ergocryptine in forages and/or grains. Adverse effects on livestock performance are typically observed when total dietary ergopeptine alkaloids exceed 100 to 200 parts per billion (ppb) [3,28]. Fescue toxicosis generally represents the effects of sub-acute to chronic exposures to 200 to 600 ppb of ergovaline commonly found in fescue grass pastures infected with endophytes [28]. Concentrations of 150 ppb were associated with reduced growth in lambs, and fescue toxicosis in heat-stressed steers was reported when ergovaline concentrations were approximately 200 ppb in the total feed [36]. Oregon State University researchers have reported higher dietary ergovaline thresholds concentrations, specifically related to the induction of clinical disease, in horses (300 to 500 ppb), cattle (400 to 750 ppb) and sheep (500 to 800 ppb) [19]. However, agalactia and clinical fescue toxicosis has also been reported in mares that were fed total dietary concentrations of ergovaline as low as 50 ppb and 100 ppb, respectively [36]. Mares (n = 4) grazing endophyte-infected fescue pasture, with relatively low serum ergovaline concentrations between 0.7 to 3.8 pg/ml (the concentration of other ergot alkaloids was not reported), displayed clinical signs of ergot alkaloid reproductive toxicity including agalactia, prolonged gestation, placental thickening, mare and neonatal mortality [37]. Ergovaline concentrations may vary within and between different fields of the same grass variety, even with the same level of infection, at different times of the year and in different years. It is important to note that increasing levels of nitrogen fertilizer on pasture can lead to an increase in ergovaline concentrations, as ergovaline is produced when plants are growing under stress [19].

### Ergot alkaloids in Ireland

Little data are currently available about ergot alkaloid in Ireland, with reports currently confined to ergotism caused by *Claviceps purpura*. This may be explained, at least in part, by the recognised association between ergot alkaloid intoxication and tall fescue (*F. arundinacea*) grasses, which are much less common in Irish pastures than in pastures in other parts of the world. In Ireland, there is a predominance of perennial rye grass, Italian ryegrass (*L. multiflorum*) and white clover (*Trifolium repens*), with perennial rye grass alone accounting for 95% of the forage grass seed sales in Ireland [38]. *Neotyphodium* species were identified in 11 of 28 wild seed populations of perennial ryegrass collected in Ireland [39] during 1981, however, the species of the endophytic fungus was uncertain.

Case reports from Ireland, with suspected ergot alkaloid involvement, are presented in Table 2, and available evidence of ergot alkaloid in herbage in Ireland in Table 3. Herbage ergovaline concentrations reported in Irish samples are not dissimilar to concentrations that are associated with adverse sub-clinical effects on livestock performance in North America [3,29]. In some of the seed samples analysed, ergovaline concentrations may have been sufficient to induce clinical ergot alkaloid intoxication.

**Table 2:** **Cases of suspected ergot poisoning in cattle in Ireland, from spring 1986**[9]

**Animal species**	**Presentation**	**Notes**
2½ year old Friesian heifer	Very dull and inappetant, obvious pinging on auscultation and percussion of the left paralumbar fossa associated with a large empty rumen, marked ketonuria	Fed mouldy concentrates, aborted at 8 months gestation
5 year old Jersey cow	Recumbent, *in extremis*	Silage fed
1 year old Friesian bullock	Quite bright, eating normally	Silage fed
3 month old Friesian calf	Quite bright, eating normally	Fed poor quality hay

All four animals were negative on both serology and bacteriology to *Salmonella* spp. All animals presented with cold insensitive extremities, and with some degree of sloughing of the ear tips, tail and digital horn of the hind limbs.

**Table 3:** Ergot alkaloid in herbage from Ireland (unpublished reports from the Irish Equine Centre, samples were submitted to either the veterinary diagnostic laboratory, Iowa State University, USA or the veterinary medicine diagnostic laboratory, University of Missouri Veterinary Medicine, USA, for determination of ergovaline by HPLC)

**Plant samples**	**Date, number**	**Ergovaline concentration (ppb)**	**Notes**
Herbage samples from perennial ryegrass (*Lolium perenne*)-rich pastures	April 2007, n = 39 (including 5 pooled samples)	10 - 210 (for 17 samples, all other samples were below the limit of detection)	Collected from a cattle farm in Co. Kilkenny, with long-term problems of poor performance, and from the wider area at two distinct time points as part of a trace element survey [40]
	September 2007, n = 25 (including 5 pooled samples)	20 - 75 (for 8 samples, all other samples were below the limit of detection)	
Herbage	May-October 2010, grass (n = 12) and haylage (n = 3)	<25 - 300	Collected on 13 stud farms in counties Kildare, Kilkenny and Cork where veterinary surgeons reported high incidences of infertility, endometrial oedema, red bag presentations, post partum hemorrhage in mares, rotaviruses outbreaks in 2 to 3 month old foals at grass, and distal leg oedema in yearlings. Some conditions were reported to be responsive to changes in herbage or topping of ergotised seed heads
Perennial ryegrass seed	May-June 2010, n = 8	<25 - 760	Seed from commercial suppliers
Perennial ryegrass/clover seed mixes	May-June 2010, n = 3	<25, 100 and 1420	
Fescue grass seed mix	May-June 2010, n = 1	900	
Timothy grass seed	May-June 2010, n = 2	<25	

## Conclusions

Given that ergovaline, typically associated with tall fescue grasses has been reported in perennial ryegrass in Ireland, and was associated with poor performance in animals, there may be a need to:

• Confirm the fungal source of the ergot alkaloids so far identified in Ireland;

• Undertake epidemiological investigations to determine the temporal and spatial distribution of ergot alkaloid intoxication of Irish perennial ryegrass;

• Develop a coordinated national sampling, testing and reporting system for ergot alkaloid in forages and feeds; and

• Consider the need for inclusion of ergot alkaloid testing within the EU seed certification regulatory framework.

Pending additional information, these results suggest that Irish farm advisors and veterinarians should be cognisant of the potential adverse role that ergot alkaloid from infected pasture grass can have on animal health and performance.

## Competing interests

The authors declare they have no competing interests.

## Authors’ contributions

MJC, UF, MKS and SJM conceived the review and participated in its design. MJC prepared the manuscript, with considerable input from UF, MKS and SJM. All authors read and approved the final manuscript.
